# The Impact of an Online Crowdsourcing Diagnostic Tool on Health Care Utilization: A Case Study Using a Novel Approach to Retrospective Claims Analysis

**DOI:** 10.2196/jmir.5644

**Published:** 2016-06-01

**Authors:** Jessie L Juusola, Thomas R Quisel, Luca Foschini, Joseph A Ladapo

**Affiliations:** ^1^ Evidation Health, Inc San Mateo, CA United States; ^2^ New York University School of Medicine Departments of Medicine and Population Health New York, NY United States

**Keywords:** crowdsourcing, diagnosis, mHealth, online systems

## Abstract

**Background:**

Patients with difficult medical cases often remain undiagnosed despite visiting multiple physicians. A new online platform, CrowdMed, uses crowdsourcing to quickly and efficiently reach an accurate diagnosis for these patients.

**Objective:**

This study sought to evaluate whether CrowdMed decreased health care utilization for patients who have used the service.

**Methods:**

Novel, electronic methods of patient recruitment and data collection were utilized. Patients who completed cases on CrowdMed’s platform between July 2014 and April 2015 were recruited for the study via email and screened via an online survey. After providing eConsent, participants provided identifying information used to access their medical claims data, which was retrieved through a third-party web application program interface (API). Utilization metrics including frequency of provider visits and medical charges were compared pre- and post-case resolution to assess the impact of resolving a case on CrowdMed.

**Results:**

Of 45 CrowdMed users who completed the study survey, comprehensive claims data was available via API for 13 participants, who made up the final enrolled sample. There were a total of 221 health care provider visits collected for the study participants, with service dates ranging from September 2013 to July 2015. Frequency of provider visits was significantly lower after resolution of a case on CrowdMed (mean of 1.07 visits per month pre-resolution vs. 0.65 visits per month post-resolution, *P*=.01). Medical charges were also significantly lower after case resolution (mean of US $719.70 per month pre-resolution vs. US $516.79 per month post-resolution, *P*=.03). There was no significant relationship between study results and disease onset date, and there was no evidence of regression to the mean influencing results.

**Conclusions:**

This study employed technology-enabled methods to demonstrate that patients who used CrowdMed had lower health care utilization after case resolution. However, since the final sample size was limited, results should be interpreted as a case study. Despite this limitation, the statistically significant results suggest that online crowdsourcing shows promise as an efficient method of solving difficult medical cases.

## Introduction

Patients experiencing complex, non-specific medical symptoms often are misdiagnosed or go undiagnosed for years [1,2]. This problem leads to avoidable health care costs from unnecessary diagnostics and treatments that occur prior to accurate diagnosis, as well as increased patient morbidity and mortality from both unnecessary procedures and treatments, as well as underutilization of appropriate care [3-7]. Many patients visit multiple physicians seeking an accurate diagnosis, but their access to care is typically limited to their geographic area and health insurance network, as well as by their time availability. Several services seek to expand access to medical care through second-opinion programs, but these programs do not involve interaction with the patient, beyond a review of their medical records, and are limited to one medical expert’s opinion [8,9].

With the recent focus on digital innovation in health care, new tools are being developed to facilitate more efficient diagnoses. Various online or mobile phone app-based symptom checkers that use computer algorithms to facilitate self-diagnosis are freely available [10]. However, these tools are intended for fairly typical patient presentations rather than the highly nuanced nature of complex, difficult to diagnose medical cases. Other tools aim to make the second-opinion process more efficient and accessible by bringing it online. One unique new tool specifically focuses on helping patients with difficult medical cases obtain an accurate diagnosis. CrowdMed is an online platform that uses crowdsourcing to overcome the limitations of regional provider access and second-opinion services (CrowdMed, Inc., San Francisco, CA). Crowdsourcing has been proven to be valuable in various fields, for everything from simple to complex tasks, including map generation, logo creation, disease prevalence estimation, and medical image categorization [11-14]. CrowdMed applies the crowdsourcing concept to diagnosis of difficult medical cases by making patient case details available to large groups of people interested in helping solve the case. Patients submit a case containing symptoms, medical history, family history, imaging, and other information regarding their disease. Registered *medical detectives* review the case and suggest potential diagnoses, and a prediction algorithm identifies the most probable diagnosis. More details on CrowdMed can be found on the company website or in Meyer et al. [15].

Meyer et al. previously conducted an independent analysis of the data collected through CrowdMed as an initial assessment of its impact [15]. This assessment was based on self-reported data collected in patient questionnaires as part of the CrowdMed experience. As such, the authors conclude that further empirical validation of the platform’s impact is necessary. To analyze its effect on health care utilization, we sought to conduct a more rigorous assessment of the impact of CrowdMed by using medical claims data from patients who completed a case on CrowdMed. Specifically, we explored the hypothesis that using CrowdMed can lower health care utilization by shortening the pathway to an accurate diagnosis. We employed technology-enabled clinical study methods to access and collect retrospective patient-specific medical claims data from a broad range of health insurers across the United States.

## Methods

### Study Overview

Just as digital innovation has led to new health care tools, it has created opportunities for new study methodologies and processes [16]. In this study, we electronically recruited and enrolled patients who had previously used CrowdMed for a difficult medical diagnosis. We used an online survey to collect information from participants that allowed us to access and retrieve their retrospective medical claims data over a relevant time period through a third-party web application program interface (API). This study was reviewed and approved by Solutions Institutional Review Board (IRB; IRB Identifier 1JUN15-108) and all participants signed informed consent documentation, as well as a Health Insurance Portability and Accountability Act authorization.

### Participants and Study Procedures

Patients who completed a case on CrowdMed’s platform between July 1, 2014, and April 30, 2015, were at least 18 years of age, and resided in the United States were identified from CrowdMed’s user database as eligible for the study. Eligibility was not based on any measure of whether the case was successful or not. Users who had submitted multiple cases were excluded from eligibility. Invitations to the study were sent via email between June 26, 2015, and July 30, 2015, with follow-up phone calls made to bolster enrollment efforts. Users who had previously unsubscribed from the CrowdMed email list did not receive a study invitation, in order to remain compliant with Controlling the Assault of Non-Solicited Pornography and Marketing Act regulations.

Study invitations included a link that brought the patients to the online study interface. Patients first read through information about the study and provided informed eConsent as well as permission to release protected health information for research. Participants were then screened to confirm that they visited at least two different physicians and/or health care facilities in the 12 months prior to using CrowdMed, where the primary reason for the visit was the illness in the CrowdMed case. These parameters were used because CrowdMed is targeted at patients who have already unsuccessfully tried to arrive at a diagnosis for their condition through the traditional health care system.

Study participants were then asked to provide identifying information, and this information was used to access their medical claims data for the 12 months prior to, and time since, their CrowdMed case resolution; a period ranging from 3 to 12 months. This information included member identification numbers and health insurer names for any health insurance plans that participants were enrolled in over that time period, as well as names and addresses for all the physicians and health care facilities they visited in that time period. Entering a national provider identifier (NPI) for each provider was optional. The time periods of 12 months prior to case resolution and 3 to 12 months since resolution were selected to construct a time period (15 to 24 months) which was long enough to capture representative health care utilization, but recent enough that patients could be expected to remember their insurance and provider information over the entire interval.

### Claims Data and Measures

Information collected from study participants was used to query a third-party web API in order to access medical claims data. The API is designed to be accessed by automated software and to return information in a consistent, software-readable format, and is hosted by a third-party company that grants access based on negotiated contracts. We wrote software to automatically query the third-party API for our study participants and quickly pull patient-specific coverage and claims information from different health insurance companies. This approach is more flexible and scalable than being limited to a costly existing claims data set that may or may not include data for patients of interest. The third-party API has wide coverage but does not give access to claims data from every health insurer in the United States, so data was not available for some study participants because of their health insurance coverage. Additionally, the third-party API required us to query it with the name and NPI of the billing provider to access claims data for the study participants; information was not always entered accurately by the study participant, likely because they were unaware of provider billing processes. We cross-referenced provider names and NPIs with others at the same practice or address, in an attempt to find the correct name and NPI for all API calls, but we were not able to pull claims data for all providers entered by participants.

Due to the limitations on data access, study participants were individually assessed for final enrollment eligibility based on the completeness of the set of medical claims accessed, in order to ensure the integrity of the study data. Participants were officially enrolled in the study if they met the following criteria: (1) claims were collected from all insurance carriers under which the respondent had coverage during the relevant time period, and (2) claims were collected from at least 67% of the health care providers entered in the study survey.

Claims data collected via the API included service date(s), Current Procedural Terminology (CPT) codes identifying medical procedures and services received, total charged amount for the claim, total amount paid on the claim by the insurer, and the provider information from the API call. This data was linked with the study participant’s CrowdMed data, including demographic information, date of case resolution, and CrowdMed questionnaire results. The combined data set was then stripped of all identifying patient information for analysis, including names, geographic information, telephone numbers, email addresses, medical record numbers, health plan beneficiary numbers, and account numbers.

The primary objective of the study was to assess whether the use of the CrowdMed service decreased health care utilization, which we hypothesized to be the case due to a shortened, more efficient diagnostic pathway with CrowdMed. This endpoint was prespecified as a decrease in monthly frequency of provider visits between the 12 months prior to CrowdMed case resolution and the time since case resolution. Provider visits were defined as one visit per date of service and unique provider (ie, claims from multiple providers on the same day qualify as multiple visits, but claims from the same provider on the same day qualify as one visit). Pharmacy claims were excluded from the visits analysis because they do not involve provider-patient interaction.

Secondary and exploratory analyses examined metrics such as decreases in monthly medical costs, frequency of high-cost medical services or procedures, and complexity of provider visits between pre- and post-CrowdMed case resolution time windows. One measure of complexity of visits was defined based on CPT codes for evaluation and management (E/M) provider visits. For example, 99213 signifies a *Level 3 Established Patient Office Visit* while code 99214 signifies a *Level 4 Established Patient Office Visit*, where higher levels denote more patient-physician face-to-face time, a more detailed and extensive exam, and more complex medical decision making. We also stratified the utilization results by patient characteristics, such as time since disease onset. For cost analysis, we used charged amount, rather than paid amount, because the amount paid out by the insurer excludes deductibles and co-insurance and therefore underestimates expenditures in a non-uniform pattern. Seasonality is especially problematic with deductibles and biases the pre- and post-time window comparisons. While charged amounts do not accurately reflect expenditures, since insurers actually pay contracted rates for each provider, they are useful for the relative comparisons conducted in this study. We removed duplicate claims for cost analysis, characterizing unique claims based on a unique combination of date of service, provider, charged amount, and CPT codes.

We also performed a comprehensive exploration of data trends over time to rule out other possible temporal confounders. To address questions of whether a period of artificially high health care utilization occurred while the patient had a case actively on CrowdMed (the *medical detectives* often request new diagnostic test results while a case is on the site), and thus biased pre-case resolution utilization as artificially high, we compared utilization metrics from the periods before initiating a case on CrowdMed, while the case was actively on CrowdMed, and post-case resolution. We also investigated whether regression to the mean could influence study results. The hypothesis in this case would be that patients initiate a CrowdMed case at a time when their symptoms are exceptionally severe, and hence the CrowdMed patient pool is biased toward patients experiencing a spike in their symptoms over patients at normal or low symptom levels. After signing up, those patients experiencing a spike in their symptoms would revert to normal symptom levels and would have been expected to improve even without intervention. The signature of this trend would be utilization that spikes right at the time of CrowdMed signup and then tapers off. To test this hypothesis, we examined the directional trend in utilization during the time frame when a case was active on CrowdMed (ie, between case initiation and resolution). A downward trend during this time period would provide evidence that regression to the mean may be present.

### Statistical Analysis

We used the one-tailed Wilcoxon signed-rank test to compare data between the pre- and post-CrowdMed case resolution time windows. We chose the Wilcoxon signed-rank test over the paired t-test because its assumptions hold for datasets with small sample sizes and values drawn from skewed distributions. The one-tailed test was used for pre-post comparisons because we were assessing whether the post-resolution utilization was or was not significantly lower than the pre-resolution utilization. When comparing the difference in pre-post utilization change between patient subgroups we used a two-tailed Wilcoxon signed-rank test, since we did not hypothesize a specific direction for a difference. For comparisons between the characteristics of the study sample and the overall population of CrowdMed users eligible for recruitment, we used the two-tailed versions of the Mann-Whitney *U* test for comparisons of continuous values, and Fisher’s exact test for comparisons of proportions.

Statistical tests were performed using R 3.2.2 (Vienna, Austria). Data processing was performed in Python 2.7.10 using the Pandas package [17]. All significance is noted at an alpha level of .05.

## Results

### Study Sample

**Figure 1:**
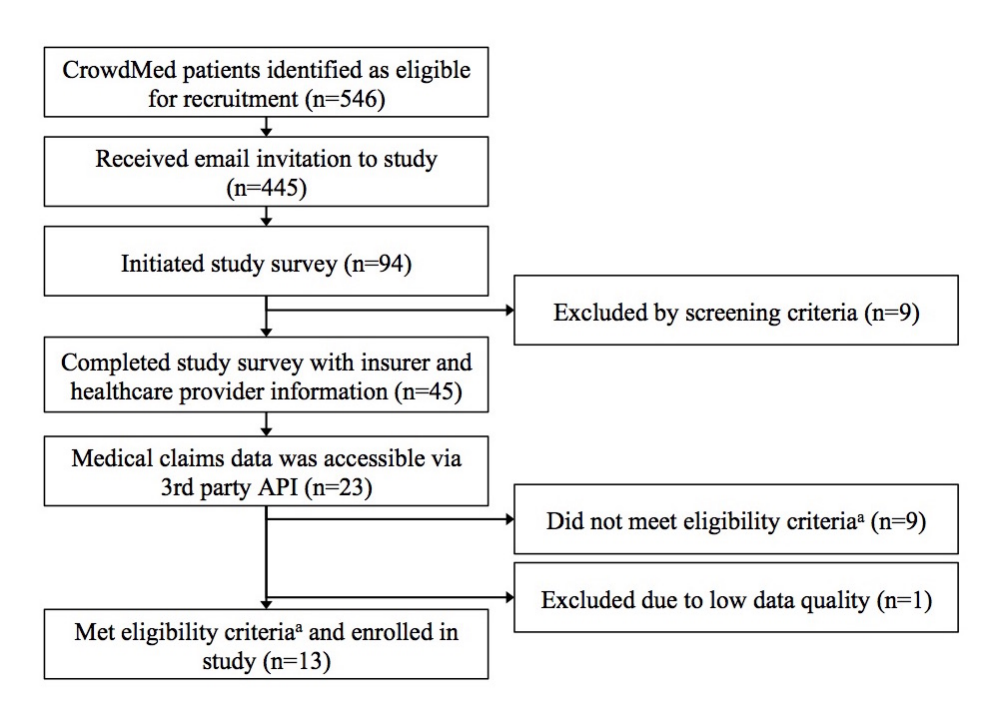
Participant recruitment and enrollment flowchart. aEligibility criteria: (1) claims were collected from all insurance carriers under which the respondent had coverage during the relevant time period, and (2) claims were collected from at least 67% of the healthcare providers entered in the study survey.

Of the 546 CrowdMed patients eligible for recruitment, 445 were sent email invitations to the study, 94 initiated the study survey, and 45 completed it with information for both their health insurer(s) and health care providers (Figure 1). Some amount of medical claims data was accessible via third-party web API for 23 patients. Of these patients, 9 did not meet enrollment eligibility criteria for completeness of their medical claims data set, and 1 was excluded due to irregular claims data of questionable quality. A total of 13 participants were enrolled in the final study sample.

Table 1 presents the characteristics of the study participants, as well as the characteristics of the entire eligible CrowdMed user population. Study participants were directionally more likely to be younger and female, compared to the eligible CrowdMed user population, though neither difference was significant. Study participants were similar to eligible users with regards to ethnicity, time since disease onset, and time since case resolution date. Most study participants had visited fewer than 10 health care providers in the 12 months prior to using CrowdMed.

**Table 1:** Participant characteristics including standard deviations (SD).

Characteristic	Study Participants (n=13)	CrowdMed Users Eligible for Recruitment (n=546)	Study Participants vs. Eligible Users, *P*-value
Mean age, years (SD)	42.8 (13.5)	51.8 (16.5)	.08^a^
Sex (%)			.28^b^
Female	9 (69%)	289 (52.9%)	
Male	4 (31%)	257 (47.1%)	
Ethnicity (%)			.66^b^
Caucasian/white	11 (85%)	483 (88.5%)	
Other	2 (15%)	63 (11.5%)	
Mean years since disease onset (SD)	6.7 (5.9)	7.6 (10.8)	.46^a^
Mean months between case resolution date and claims data pull (SD)	6.9 (2.8)	8.0 (3.1)	.22^a^
Health care providers visited in the 12 months prior to using CrowdMed, self-reported in screener (%)			N/A
2-4	6 (46%)	N/A	
5-9	6 (46%)	N/A	
10+	1 (8%)	N/A	

^a^ *P*-value of a Mann-Whitney *U* test

^b^ *P*-value of Fisher’s exact test

### Health Care Utilization

**Figure 2:**
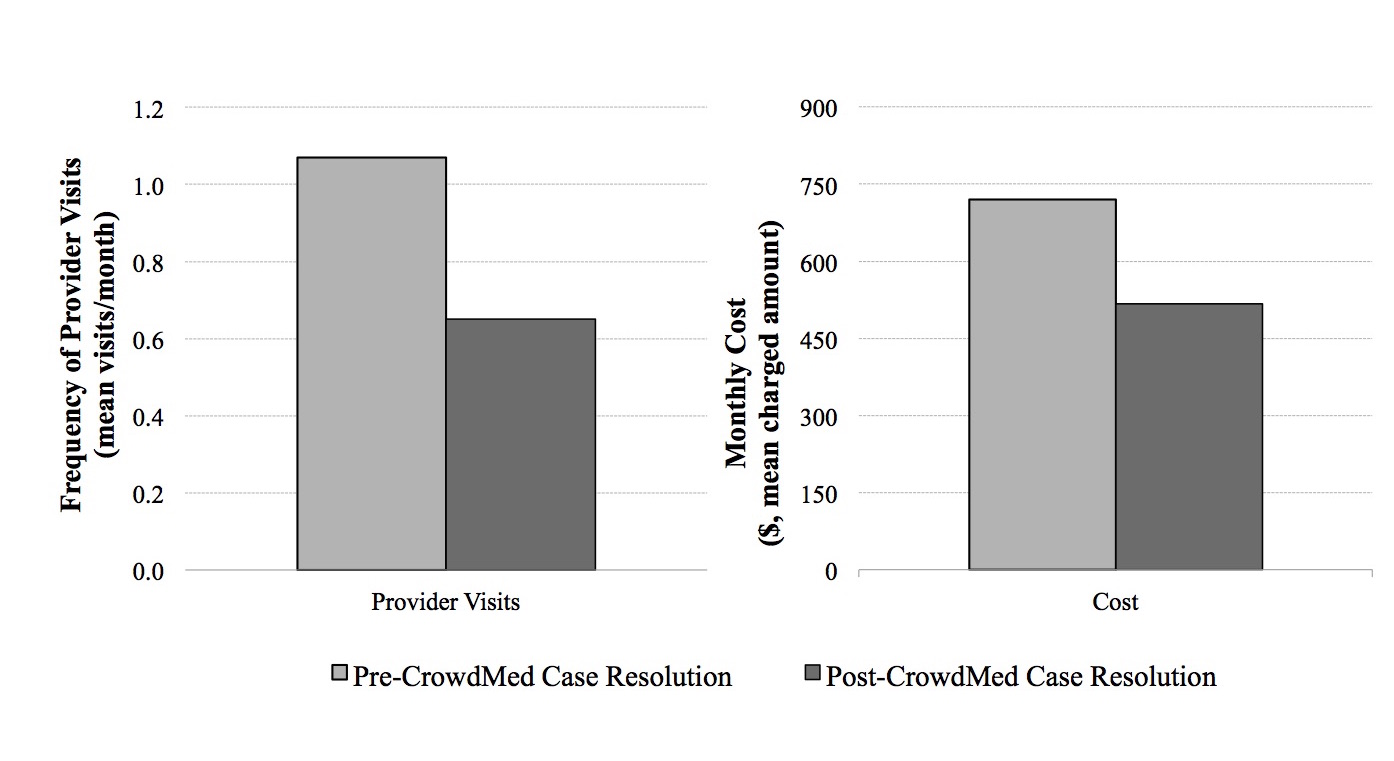
Impact of CrowdMed case resolution on healthcare utilization.

A total of 221 health care provider visits were collected for the study participants (167 prior to CrowdMed case resolution and 54 post-resolution) across 11 US health insurers, with service dates ranging from September 20, 2013, to July 28, 2015. Health care utilization was significantly lower after resolution of a case on CrowdMed compared to before case resolution. The primary endpoint of frequency of health care provider visits decreased significantly, from an average of 1.07 visits per month pre-case resolution to 0.65 visits per month post-case resolution (median visits per month 0.50 vs. 0.34, *P*=.01, *W*=79; Figure 2). Medical costs, calculated based on charged amounts as previously described, were also significantly lower after case resolution (mean of US $719.70 per month vs. US $516.79 per month, median of US $349.92 per month vs. US $60.83 per month, *P*=.03, *W*=72). Additionally, health care utilization trended downward with time from case resolution date.

We examined metrics and proxies for a decrease in complexity of care in exploratory analyses. The monthly frequency of procedures and services over US $500 was directionally lower after case resolution with a trend toward statistical significance (mean of 0.31 vs. 0.17, median of 0.25 vs. 0.00, *P*=.06, *W*=51). Number of CPT codes per visit and average cost per visit were also directionally lower post-case resolution (mean of 3.3 vs. 2.4, median of 3.1 vs. 2.0, *P*=.19, *W*=20; and mean of US $470 vs. US $400, median of US $439 vs. US $256, *P*=.13, *W*=27), as were the complexity levels of provider visits based on E/M visit CPT codes. Prior to CrowdMed case resolution, 17% of visits were coded as Level 3 office visits (intermediate complexity), while 28% were coded as Level 4 office visits (higher complexity). After CrowdMed case resolution, 24% of provider visits were coded as Level 3 office visits while 19% were coded as Level 4 office visits.

We explored stratifying the study results by various patient characteristics but did not find any factors that significantly correlated with the key result of lower frequency of provider visits post-CrowdMed case resolution. We first examined time since disease onset. Study participants were split by disease onset in 2010 or earlier (n=6) and 2011 or later (n=7). There was no significant difference in the results for change in provider visit frequency post-case resolution between the groups (*P*=1.00, *U*=22). We also incorporated a metric that CrowdMed internally uses to designate cases as successful or unsuccessful. This metric is subjective and based on a question that users answer in a survey after their case is resolved, indicating whether the user believes that CrowdMed brought them closer to a correct diagnosis or cure. In our study sample, 9 participants had responded positively while 4 had responded negatively to the self-report question (at 69%, this is similar to the results CrowdMed typically sees across their surveyed user population of 60% to 70%). There was no significant difference between the two groups in the results for change in provider visit frequency (*P*=.28, *U*=11).

**Figure 3:**
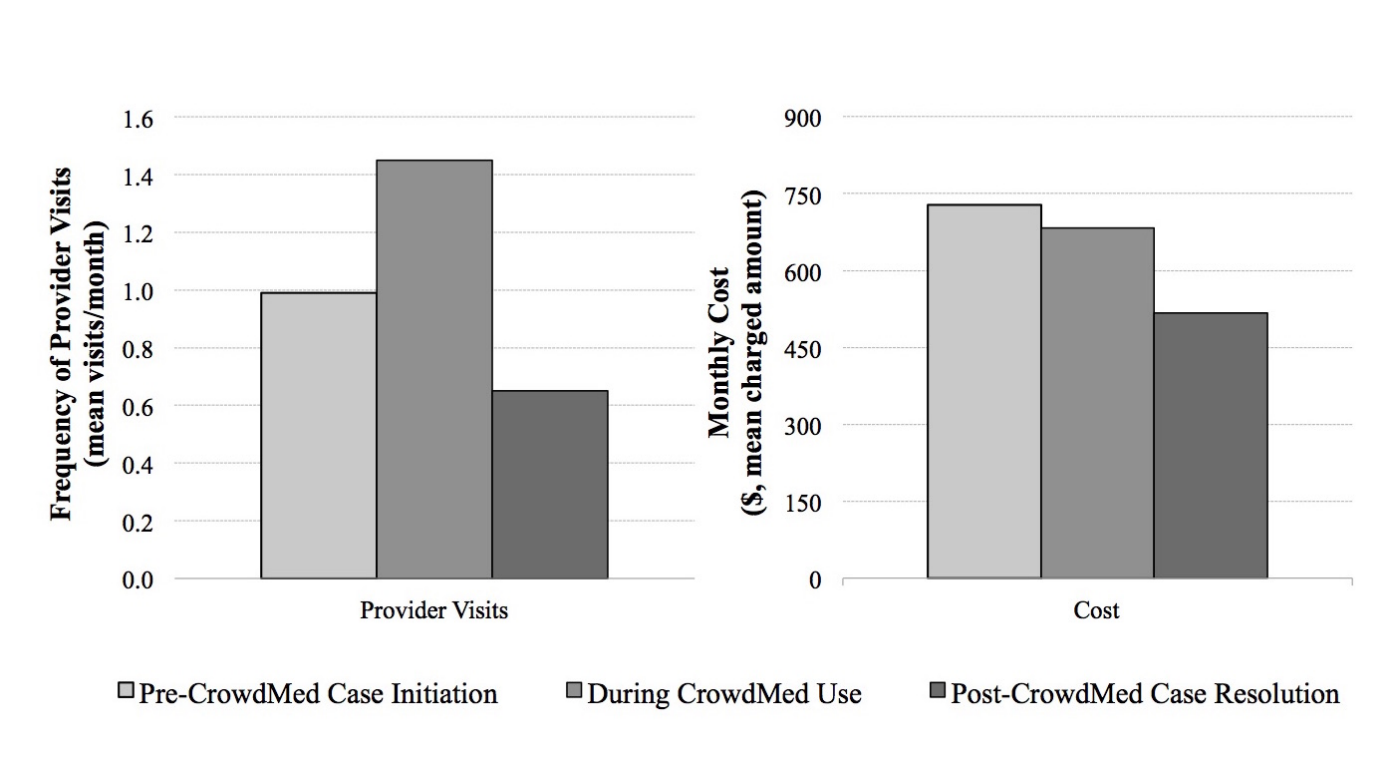
Healthcare utilization before CrowdMed case initiation, during CrowdMed use, and after case resolution.

In order to assess whether a period of artificially high health care utilization occurs while a case is active on CrowdMed, and biases pre-resolution utilization rates, we split the pre-resolution time period in two: before case initiation versus while active on CrowdMed. We compared utilization during these time periods against each other and against post-case resolution utilization. We found that monthly provider visits were significantly higher during the period that a case was active on CrowdMed compared to before case initiation (mean of 0.99 visits per month before case initiation vs. 1.45 visits per month during CrowdMed use, median of 0.40 vs. 1.00, *P*=.047, *W*=70; Figure 3). This result suggests that there is a spike in utilization while a case is active on CrowdMed. To determine if this issue biases study results, we removed the period that a case was active on CrowdMed and compared the before-case-initiation time period with the post-resolution time period. We found that monthly provider visits were significantly lower post-case resolution compared to before case initiation (mean of 0.99 visits per month before case initiation vs. 0.65 visits per month post-case resolution, median of 0.40 vs. 0.34, *P*=.02, *W*=65), which is consistent with our base-case results.

Finally, we investigated whether regression to the mean may have influenced study results (ie, patients signed up for CrowdMed when their symptoms were exceptionally severe, and their symptoms would revert to normal levels even in the absence of CrowdMed). This trend would be signified by utilization that peaks at the time of CrowdMed signup and then tapers off. To test this hypothesis, we specifically examined the time frame when a case was active on CrowdMed (between case initiation and resolution) and the directional trend in utilization during this period. A downward trend would provide evidence of regression to the mean. We found a positive correlation of 0.18 between provider visits per week and weeks since CrowdMed case initiation. The correlation between cost per week and weeks since case initiation was similar, at 0.17. This upward trend provides evidence that regression to the mean is not a major determining factor in our study results.

## Discussion

### Principal Results

This study empirically demonstrated that health care utilization is significantly lower after resolving a difficult medical case on CrowdMed, as evidenced by a small sample of previous CrowdMed users. Both monthly frequency of provider visits and monthly charges were significantly decreased after case resolution per medical claims data. Results were consistent when stratified by various patient characteristics, such as time since disease onset. Additionally, it appears that complexity of care may decrease after case resolution. While results were only directional rather than statistically significant, there were multiple indicators of health care visits and care being less complex after case resolution, such as lower frequency of high-cost procedures, fewer CPT codes per visit, and Level 3 visits becoming more likely than Level 4 visits. The relatively small sample size in the study may explain the lack of statistical significance, rather than a lack of effect.

In addition to the valuable insights that this study provides into the impact of crowdsourcing for diagnosis of difficult medical cases, it also serves as an example of how new technologies can drive innovation in clinical research implementation. Clinical studies have traditionally been limited to site-specific geographies, whereas online recruitment and data collection methods afford broad patient access. Here, we were able to access and recruit CrowdMed users irrespective of where they were located in the United States. Data access for retrospective claims analyses has also been limited in the past to insurer-specific data sets or expensive, often de-identified, data sets for purchase. This is the first study to use a web API to access patient-specific claims data across a broad range of insurers. This new method of claims data access widens the realm of possibilities for claims analyses. Claims data is an objective indicator of health care utilization, but historically it has been infeasible to collect it from patients themselves. This study demonstrates that new technology enables another effective approach to claims data access, which can be used to test the impact of emerging technologies that may not yet be identifiable through claims codes, or may not have adequate penetration to be studied in any single insurer’s separate data set.

The objective nature of a claims analysis is a strength for this study. However, the limitations of claims data may make some aspects of our findings conservative as well as uncertain in a few key manners. For example, claims data excludes quality of life factors. In this study, most participants had lower health care utilization after they resolved their case on CrowdMed. A few, however, had increased provider visit frequency and/or costs. This is not unexpected, as an accurate diagnosis can lead to proper treatment that will improve the patient’s quality of life and possibly long-term survival. This treatment could be expensive, but we would need to study the cost-effectiveness of the treatment to determine its overall value. Similarly, CrowdMed could bring a patient closer to an accurate diagnosis by placing that patient on an appropriate diagnostic pathway, which could entail expensive diagnostic tests. Although this may be reflected in our analysis as higher costs post-case resolution, it could represent tests that would be required for an accurate diagnosis, and CrowdMed may have accelerated the steps to those tests. Another conservative aspect of this study (and similar claims analyses) is the exclusion of indirect costs. Provider visits involve cost implications for missed work and travel to appointments, and these costs are borne by patients, caregivers, and employers. While these indirect costs may be irrelevant to some health care payers, they are very impactful to self-insured employers. The ability of CrowdMed to reduce the frequency of provider visits would translate directly to a more productive workforce.

### Limitations

This study had several limitations. First, the enrolled sample was small, at 13 patients. As such, we present these results as a case study to acknowledge the potential for lack of generalizability to a larger sample. Despite this, the primary endpoint of change in monthly frequency of provider visits between the pre- and post-case resolution time windows was statistically significant, as was the change in monthly cost. Second, we were limited to charged-amount data for cost analysis, since allowed or contracted amount was not available and insurer paid amount is biased by deductibles. While the relative comparisons we conducted are meaningful with charged-amount data, the absolute numbers themselves are more difficult to interpret. We can apply a modified cost-to-charge ratio that approximates insurer reimbursement rates to provide a more accurate estimate of cost. One estimate is that commercial insurer contracted rates are approximately 70% of charges [18], suggesting that costs are on average US $142 per month lower post-case resolution ($719.70 − $516.79 = $202.91, $202.91 × 70% = $142.04). Thirdly, our hypothesis that use of the CrowdMed service decreases health care utilization by shortening the pathway to an accurate diagnosis does not account for the possibility that some patients give up on the diagnostic search and thus underutilize care. For these patients, CrowdMed may be able to improve their care by increasing their utilization. This issue would be a valuable exploration for a subsequent study, as it is outside the scope of this analysis and would require data sources other than claims. Additionally, our current claims-based approach does not discriminate between health care utilization related to the illness in the CrowdMed case or for unrelated reasons. However, there is no reason that unrelated health care utilization should bias our results in one direction over another.

The pre-post study design was also limited by the lack of an external control arm. This issue prompts questions of whether results were biased by an artificial increase in health care utilization during the time a case was active on CrowdMed, or whether regression to the mean influenced results. Our analyses indicate that there is a spike in utilization while a case is active on CrowdMed. This is not surprising, because the CrowdMed *medical detectives* may ask for specific test results to guide their diagnostic suggestions. Despite this increased utilization during the active CrowdMed period, we found that monthly provider visits were still significantly lower post-case resolution compared to before case initiation, which demonstrates that study results hold when controlling for increased utilization during the active case period. As for the possibility that patients initiate a CrowdMed case at an especially intensive period of utilization and disease severity, and would be expected to improve even without intervention, our analyses suggest this is not a factor in our results. Time since disease onset varies in the sample, and results are independent of this patient characteristic, which suggests that patients enter CrowdMed at various points in their disease progression. Additionally, we found that there is an upward trend in frequency of provider visits and cost as a function of time since CrowdMed case initiation during the period the case is active, rather than the downward trend that would be expected if regression to the mean were present.

### Conclusions

In this case study, patients who resolved a difficult medical case on the CrowdMed platform were found to have approximately 40% lower frequency of provider visits and 30% lower medical charges after case resolution. This early evidence suggests that wider spread use of crowdsourcing diagnoses in difficult medical cases may have the potential to reduce the burden on the US health care system and lead to more efficient delivery of health care resources. These findings can serve as an initial value assessment for potential purchasers of CrowdMed services and can serve to inform subsequent larger, prospective, controlled studies that bolster the evidence of CrowdMed’s impact.

## Abbreviations

API: application program interface
CPT: Current Procedural Terminology
E/M: evaluation and management
IRB: Institutional Review Board
NPI: national provider identifier
SD: standard deviation
